# “Unreasonable” ventricular pacings

**DOI:** 10.1111/anec.12793

**Published:** 2020-08-21

**Authors:** Yubin Zhang, Tong Liu, Panagiotis Korantzopoulos

**Affiliations:** ^1^ The First Affiliated Hospital School of Medicine Zhejiang University Hangzhou China; ^2^ Tianjin Key Laboratory of Ionic‐Molecular Function of Cardiovascular Disease Department of Cardiology Tianjin Institute of Cardiology Second Hospital of Tianjin Medical University Tianjin China; ^3^ First Department of Cardiology University Hospital of Ioannina Ioannina Greece

**Keywords:** electrocardiography, implantable devices, noninvasive techniques, pacemaker mediated tachycardia

## Abstract

A 66‐year‐old man, implanted Abbott dual‐chamber pacemaker, was admitted to our hospital due to recurrent palpitation. ECG was recorded on admission, which created a diagnostic confusion: What accounts for the appearance of the VP in the setting of a stable intrinsic atrioventricular (AV) conduction? In this case, we will focus on the logical reasoning in the analysis of Pacing ECG.

A 66‐year‐old man, implanted Abbott dual‐chamber pacemaker, was admitted to our hospital due to recurrent palpitation. Programmed parameters were as follows: DDD pacing mode, Base rate 60 bpm, Pacing/Sensed AV interval 250 ms/200 ms, Post‐ventricular atrial refractory period (PVARP) 275 ms, Ventricular intrinsic preference (VIP) On, and VIP extension 100 ms.

Figure 1 was recorded on admission, which created a diagnostic confusion: Normally, ventricular pacing (VP) had no reason to appear in the setting of a stable intrinsic atrioventricular (AV) conduction. What accounts for the appearance of the VP?

**Figure 1:**
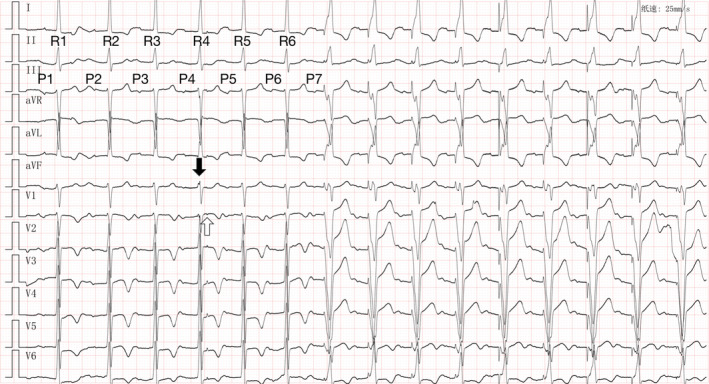
The 12‐lead ECG demonstrates the unreasonable appearance of atrial‐tracked ventricular pacing under a stable intrinsic atrioventricular conduction

## 1 EXPLANATIONS

Intermittent atrial undersensing appears to explain the confusion, but clues at R4 may not support it. There are spike‐like signal (S1, black arrow in Figure 1) occurring on R4 and another spike‐like signal (S2, hollow arrow in Figure 1) occurring on the subsequent ST segment in 100 ms. Potential explanations include (a) interference signals; (b) VP (S1)—premature atrial contraction (PAC, S2); (c) VP (S1)—ventricular pacing backup (VPb, S2); and (d) AP (S1)—ventricular safety pacing (VSP, S2). What is the diagnosis?

**Figure 2:**
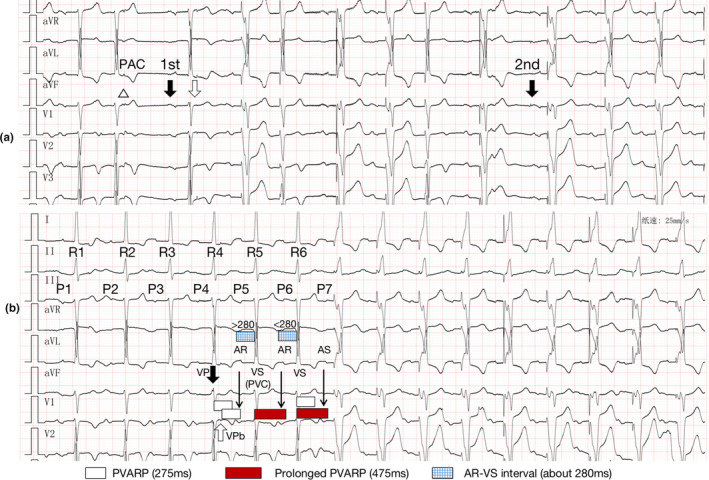
(a) The figure has the same S1–S2 sequence (hollow arrow) as it in Figure 1, and a blocked PAC (hollow triangle); (b) the deductive reasoning to explain Figure 1

Figure 2a (obtained 20 min later) demonstrated two critical clues: First, comparing the P waves below the first and the second black arrow, we can find the first was actually generated by an AP which was not recorded. And the same S1–S2 sequence was occurring on the 3rd QRS complexes (hollow arrow). Now, we have AP‐S1–S2 sequence. AP‐S1 is about 350ms. The subsequent SAV/PAV intervals are fixed at 200 ms/250 ms (some were slightly extended due to the Maximum tracking rate). Second, there is a blocked PAC (hollow triangle), the form of which was different from the S2. So (3).VP(S1)‐VPb(S2) is the only choice. In Abbott, It will suspend the VIP when the device detects a ventricular non‐capture (Bradycardia & Tachycardia). In Figure 2a (hollow arrow), the device actually detected a non‐capture due to the false fusion beat (the intrinsic QRS falling in the ventricular blanking period after VP, S1), generated VPb (S2) in 100 ms and suspended VIP.

However, the VP‐VPb in Figure 1 is occurring on R4 while the shortening of the AV interval is following the R6. Here is a 3‐Step deductive reasoning to explain (Figure 2b):

**Step 1—P7**: The first VP is generated due to the P7, which suggests that P7 is occurring outside the PVARP of R6 (white bar) and marked as AS.

**Step 2—P6 and R6**: As the R6‐P7 interval (about 320ms) is longer than 275ms and less than 475ms (PVC response prolongs the PVARP by 200ms), the R6 cannot be marked as PVC, or P7 would be marked as atrial refractory event (AR). In Abbott, a VS in AR‐VS sequence will be marked as PVC when AR‐VS interval is longer than 280ms. Therefore, AR(P6)‐VS(R6) interval (grid bar) should be less than 280ms (Of noted, PR intervals in Figure 1 are all about 280ms, so the actual AR‐VS interval can be a little bit less or longer than 280ms due to the device measuring error) to guarantee R6 not marked as PVC.

**Step 3—VPb, P5, and R5**: R5 should be marked as PVC to prolong PVARP (red bar) to guarantee P6 marked as AR (R5‐P6 interval 320ms). The only sequence that fits the condition is AR(P5)‐VS(R5), and the P5‐R5 interval should be longer than 280ms. To guarantee AR(P5), the key point is VP‐VPb in R4. VPb recycles PVARP (white bar) which results in P5 falling in the PVARP and marked as AR, and AR(P5)‐VS(R5) interval (grid bar) can be longer than 280ms due to the measuring error. These coincidences (1. VIP, 2. PVC response, and 3. VPb) lead to the “Unreasonable” Ventricular Pacings.

Inappropriate continuous ventricular pacing can cause adverse outcomes due to (a) atrioventricular asynchrony and (b) increased proportion of right ventricular pacing, which in turn cause ventricular filling and decreasing ejection volume and exacerbating heart failure (Patel & Mariani, 2017).

Extending AV interval or VIP extension can avoid such inappropriate ventricular pacings. After extending VIP extension from 100 to 150 ms, the above situation did not occur. This case should be distinguished from intermittent atrial undersensing, but there is no clear evidence to support it in Figures 1 and 2a, and the subsequent device programming showed no abnormalities in atrial sensing.

## 2 CONFLICT OF INTEREST

The authors declared that they have no conflicts of interest to this work.

## AUTHOR CONTRIBUTION

All authors reviewed and approved the manuscript. Directed this study: Yubin Zhang, Tong Liu. Wrote the manuscript: Yubin Zhang. Gave suggestions on this study: Tong Liu, Panagiotis Korantzopoulos.

## ETHICAL APPROVAL

The study was approved by the institutional review board of The First Affiliated Hospital, School of Medicine, Zhejiang University, Hangzhou, China. The patient provided written informed consent.
